# Meningococcal W135 Disease Vaccination Intent, the Netherlands, 2018–2019

**DOI:** 10.3201/eid2607.191812

**Published:** 2020-07

**Authors:** Marion de Vries, Liesbeth Claassen, Margreet J.M. te Wierik, Feray Coban, Albert Wong, Danielle R.M. Timmermans, Aura Timen

**Affiliations:** National Institute for Public Health and the Environment (RIVM), Bilthoven, the Netherlands (M. de Vries, L. Claassen, M.J.M. te Wierik, F. Coban, A. Wong, A. Timen);; Vrije Universiteit Amsterdam, Amsterdam, the Netherlands (D.R.M. Timmermans, A. Timen)

**Keywords:** meningococcal disease, IMD, health behavior, vaccination, perception, knowledge, attitude, communication, disease outbreaks, adolescent, teenager, parents, mental models, bacteria, the Netherlands, Neisseria meningitidis, W135, vaccines

## Abstract

To control the rise in *Neisseria meningitidis* strain W infections, during 2018–2019, the Netherlands launched a catch-up meningococcal conjugate (MenACWY) vaccination campaign for teenagers (13–18 years of age). Applying a mental models approach, we surveyed teenagers and their parents about their knowledge and beliefs about meningococcal disease, the MenACWY vaccination, vaccinations in general, and their MenACWY vaccination intentions. Using random forest analysis, we studied predictions of vaccination intentions by knowledge and beliefs. Survey response rate was 52.8% among teenagers and 59.4% among parents. MenACWY vaccination intentions were best predicted by knowledge and beliefs about vaccinations in general, surpassing knowledge and beliefs about meningococcal disease and the MenACWY vaccination. For teenagers, their parents’ intention that the teenager be vaccinated was a strong predictor of the teenagers’ own vaccination intention. To optimize vaccination uptake during future outbreaks, we recommend that communications emphasize the effectiveness and safety of vaccines and continue to focus on parents.

Since the end of 2015, invasive meningococcal disease (IMD) caused by *Neisseria meningitidis* strain W135 has emerged as a severe threat to public health in the Netherlands (*1*). Before 2015, IMD W135 cases occurred sporadically, averaging 4 cases per year. From 2015 on, the number of cases increased rapidly, to 103 patients in 2018 alone. Cases were reported among persons in all age groups, but the largest numbers of cases were among children <5 years of age, teenagers, and elderly persons. The case-fatality rate has been highest among teenagers/young adults 14–24 years of age (29% compared with an average case-fatality rate of 17%) (*2*).

In September 2017, the Ministry of Health, Welfare and Sport in the Netherlands decided to introduce the meningococcal conjugate (MenACWY) vaccine into the National Immunization Program for children 14 months of age (replacing the meningococcal C conjugate vaccine used until then) and to have an additional catch-up MenACWY vaccination campaign in 2018 and 2019 that focused on teenagers. The initial target groups for the catch-up vaccination campaign were teenagers ≈14 years of age (born after April 2004 and in 2005). In July 2019, because of increased vaccine accessibility, the target groups were extended to include all teenagers 14–18 years of age (cohorts 2001–2005).

Various studies have been performed to determine how persons make vaccination decisions, including those regarding vaccination against meningococcal disease (*3*–*12*). This academic interest in vaccination decisions has increased over the past few years after the gradual decline of vaccination uptake observed in many countries. Various factors play a role in this decline (*13*). Frequently mentioned causes are the lack of laypersons’ familiarity with the severe consequences of vaccine-preventable diseases, increased concerns about the safety of vaccines, and decreased trust in the effectiveness of vaccines.

In contrast to most studies that have shown the influence of knowledge and beliefs on vaccination decisions (e.g., those applying the theory of planned behavior [*14*,*15*], the protection motivation theory [*16*,*17*], and the health belief model [*18*,*19*]), our aim with this study was not to fully understand vaccination behavior but to gain insights into specific aspects of knowledge and beliefs that could provide concrete input for communication practices. Studies of vaccination decisions that apply behavioral models often study risk and benefit perceptions in relatively general terms (e.g., perceived vulnerability, severity, and safety). At the same time, the need to assess context-specific knowledge and beliefs when studying human behavior has been strongly emphasized (*20*). These specific beliefs are not only likely to better predict vaccination behavior (*20*), but insights into these beliefs can also provide more concrete input for communication (*21*).

The mental models approach, a method developed to improve risk communication in the field of environmental risks (*22*–*26*), focuses on assessing and comparing experts’ and laypersons’ knowledge and beliefs about risks. This approach has been infrequently applied in the field of infectious diseases and vaccinations (*26*–*28*). The concept of mental models suggests that persons have mental representations of risks, consisting of a complex interconnected web of both specific and more general knowledge and beliefs about the causes, effects, and risk mitigation options of that risk (*21*). Laypersons’ mental models often differ largely from those of experts, which is one reason why experts’ risk communications often do not have the desired effect among laypersons (*21*). Following the mental models approach, communications should be based not only on what experts consider important but also on what laypersons consider important and what they already know and believe. Communications thus need to be compatible with the mental model of the receiver, should correct misbeliefs, should add information that was previously lacking, and should be delivered in language that laypersons understand.

On the basis of the mental models approach, at the onset of the MenACWY vaccination campaign in September 2018, we explored aspects of knowledge and specific beliefs about meningococcal disease, the MenACWY vaccination, and vaccines in general among teenagers in the Netherlands invited for the MenACWY vaccination and their parents. We also investigated which of these aspects of knowledge and specific beliefs are strong predictors of MenACWY vaccination intentions. Those aspects of knowledge and specific beliefs that strongly predict vaccination intentions could be prioritized in future communications.

Two research questions were central to our study: What do teenagers and their parents know and believe about meningococcal disease, the MenACWY vaccination, and vaccinations in general? Which aspects of knowledge and specific beliefs predict MenACWY vaccination intentions by teenagers and their parents?

## Methods

### Study Population and Procedure

During September 13–26, 2018, we sent surveys to teenagers ≈14 years of age (born after April 2004 and in 2005; n = 1,923), to whom the MenACWY vaccination was initially directed; to their parents (n = 2,000); and to teenagers from the extended target group (teenagers born from 2001 through April 2004; n = 1,113) and their parents (n = 1,002). The surveys were conducted via an online survey panel (Kantar Public, http://www.niipo.nl/panel). At the time of this study, this survey panel had an active population of ≈140,000 residents in the Netherlands. Panel members gave active consent for their participation in the panel organization, including consent for data sharing.

Before actively entering the survey, all panel members invited to participate were informed about the purpose and content of the study. Teenagers’ participation required additional consent from 1 of their parents. Survey completion took 15 minutes on average. The Clinical Expertise Centre RIVM determined that this research was not subject to law in the Netherlands for medical research involving human subjects and, therefore, concluded that it was exempt from needing further approval from an ethics research committee.

### Survey Development

In line with the mental models approach (*21*,*29*), we based the survey questions on basic information provided by the National Institute for Public Health and the Environment (RIVM) (*2*) and on knowledge and beliefs among teenagers and parents, which we explored with open-ended, semistructured interviews. We interviewed 12 teenagers and 10 parents during April–June 2018. The interviews started with open-ended questions about vaccinations and infectious diseases in general (e.g., “What can you tell me about vaccinations?”) and consequently narrowed down to meningococcal disease and the MenACWY vaccination. In addition to the questions yielded from the RIVM information and the interviews, we supplemented the survey with questions about the safety and effectiveness of vaccines in general, derived from vaccine-skeptic websites in the Netherlands (*30*,*31*), to examine whether beliefs that contradict the RIVM information were present in the population.

### Operationalization of Concepts

The survey questions addressed MenACWY vaccination intention and various aspects of knowledge and beliefs about meningococcal disease, the MenACWY vaccination, and vaccinations in general. MenACWY vaccination intention was assessed with the question “Do you want to be vaccinated against meningococcal disease type A, C, W, and Y?” for the teenagers and “Do you want your child to be vaccinated against meningococcal disease type A, C, W, and Y?” for parents. Respondents could answer on a 7-point semantic scale, from 0 (certainly not) to 6 (certainly yes).

We assessed aspects of knowledge and specific beliefs about meningococcal disease, the MenACWY vaccination, and vaccinations in general with 5 questions, including 42 items (Appendix). Most items assessing knowledge and beliefs were formulated as statements. Respondents were asked to indicate on a 5-point Likert scale whether they thought that these statements were true or false. Exceptions to the use of this scale were items assessing respondents’ familiarity with various terms used for meningococcal disease (3-point scale) and items assessing respondents’ beliefs about short-term adverse events of vaccinations (7-point scale).

### Statistical Analyses

We performed descriptive analyses for each measure within the samples of parents and teenagers. We used independent Student *t*-tests tests to study differences between parents and teenagers with regard to MenACWY vaccination intention and knowledge and belief items, measured on 5- and 7-point Likert scales. Differences between parents’ and teenagers’ knowledge and belief items measured on a 3-point scale were studied by using χ^2^ tests.

To study whether and how knowledge and beliefs predict MenACWY vaccination intentions, we applied random forest analyses (RF) (*32*) in R (*33*). RF is a nonparametric machine learning method for regression and classification based on an ensemble of decision trees. We considered RF to be appropriate because our study has a dependent variable (MenACWY vaccination intention) that is not normally distributed, a relatively large number of (partly intercorrelated) independent variables (knowledge and beliefs), and potentially nonlinear relationships between independent variables and the dependent variable.

We built separate RF models for teenagers and parents and built a third model for all teenagers in the sample for whom at least 1 parent also participated in the survey. In this model, the knowledge and beliefs variables and the dependent variable from the parents were added as independent variables to their children’s model to study the interrelatedness of paired parents and children. All analyses were controlled for age, sex, education, income, social class (based on income, education, and employment), region of residence, teenager’s vaccination record, whether the respondent was aware of the MenACWY vaccination campaign, and whether teenagers and their parents were part of the first target group (cohort born after April 2004 or in 2005) or the second target group (cohorts born 2001–2003 or before May 2004). We used the RF method to generate 4 types of output: 1) the variable importance ranking, which ranks the independent variables in terms of how much they contribute to the explanation of the dependent variable; 2) the marginal means (MM), which describe the relationship between the dependent variable and each independent variable; 3) the total explained variance of the model; and 4) the cumulative variance explained (CVE), which indicates how much each independent variable adds to the explained variance of the model when the independent variables are added to the model following the sequence of the variable importance matrix.

## Results

### Study Population

Response rates were 52.8% among teenagers (n = 1,603/3,036) and 59.4% among parents (n = 1,784/3,002). The sample contained 1,318 pairs of a parent and a teenager from the same household (Table).

**Table:** Description of participants in study of invasive meningococcal W135 disease vaccination intent, the Netherlands, 2018–2019*

Participant	No. (%)
Sex	
Parents, n = 1,784*	
F	991 (55.5)
M	793 (44.5)
Teenagers, n = 1,603*	
F	810 (50.5)
M	793 (49.5)
Age, y†	
Teenagers	
12	111 (6.9)
13	611 (38.1)
14	379 (23.6)
15	175 (10.9)
16	161 (10.0)
17	166 (10.4)
Education‡	
Parents	
Low	252 (14.1)
Intermediate	1,318 (73.9)
High	214 (12.0)
Teenagers	
No current education	8 (0.5)
Primary school	12 (0.7)
Secondary school	1,406 (87.7)
Preparing for vocational education	552 (39.3)
Preparing for higher education	809 (57.5)
Combination	45 (3.2)
Vocational education	129 (8.0)
Higher education	48 (0.7)
Initial target group, born after Apr 2004 through 2005	
Parents	1,177 (66.0)
Teenagers	1,010 (63.0)
Extended target group, cohorts born 2001 through Apr 2004	
Parents	607 (34.0)
Teenagers	593 (37.0)

*Total parent–teenager pairs = 1,318 (73.9% of parents, 82.2% of teenagers).  †Parents’ age range 31–73 y; mean (± SD) age 46.5 (5.5) y. ‡Categorizations based on (*34*)..

### MenACWY Vaccination Intention

Teenagers were generally willing to be vaccinated with the MenACWY vaccine, and their parents were willing to have them vaccinated. Mean (± SD) scores were 5.0 (± 1.5) for parents and 4.4 (± 1.7) for teenagers. Parents were significantly more willing to have their teenagers vaccinated with the MenACWY vaccine than were teenagers willing to be vaccinated (p<0.001).

### Knowledge and Beliefs about Meningococcal Disease

Whether respondents were familiar with meningococcal disease depended on the terms used to identify it. Respondents were most familiar with the Dutch lay terms for septicemia and meningitis (Appendix). Less well known were the more expert terms for meningococci, meningococcal disease, septicemia, and meningitis. Parents were significantly more aware than teenagers of all terms used for meningococcal disease.

The average responses to most items assessing knowledge and beliefs about meningococcal disease reflected modest levels of knowledge and are mostly close to the scale median representing the “don’t know” category (Appendix). Respondents generally seemed aware of the transmissibility and seriousness of IMD and of the current outbreak, reflected by the scores on the following items: “Meningococcal disease is contagious,” “Meningococcal disease requires hospitalization for treatment,” and “In the past couple of years, more people in the Netherlands fell ill due to one of the meningococcus types.” Parents were significantly more knowledgeable than teenagers with regard to all but 1 item representing true or false statements about meningococcal disease.

### Knowledge and Beliefs about MenACWY Vaccination

In general, respondents indicated knowing that MenACWY vaccination does not confer lifelong protection against meningococcal disease (Appendix). Less well known was the fact that this vaccine does not protect against all meningococcal serogroups. Parents were significantly more knowledgeable than teenagers about the continued possibility of contracting meningococcal disease after vaccination and about the reasons why teenagers were invited to receive MenACWY vaccination.

### Knowledge and Beliefs about Vaccinations in General

On average, parents and teenagers believed that vaccinations are needed to prevent infectious diseases and are effective at doing so (Appendix). We found some misbeliefs concerning the safety of vaccines. The most prominent misbelief was represented by the relatively high scores for “Every year, a number of children in the Netherlands die from the harmful consequences of vaccines.” We did not observe a clear pattern between parents and teenagers in knowledge and beliefs about vaccinations in general; for some items, parents seemed more knowledgeable, but for others, teenagers seemed to know more. Of all short-term adverse events, teenagers were significantly more concerned than their parents about the pain caused by vaccination and less concerned about the possibility of a swollen arm after vaccination.

### MenACWY Vaccination Intentions among Teenagers and Parents as Predicted by Aspects of Knowledge and Specific Beliefs

RF analyses included all respondents who reported the teenager not having received, or not remembering having received, the MenACWY vaccination before their participation in the survey (1,541 teenagers and 1,712 parents). The RF models explain 47.2% of the variance in MenACWY vaccination intentions for parents and 31.7% for teenagers. The combined model (in this model, the knowledge and beliefs items and the dependent variable from the parents were added to their children’s models) explains 39.9% of the variance in MenACWY vaccination intentions among teenagers.

**Figure 1:**
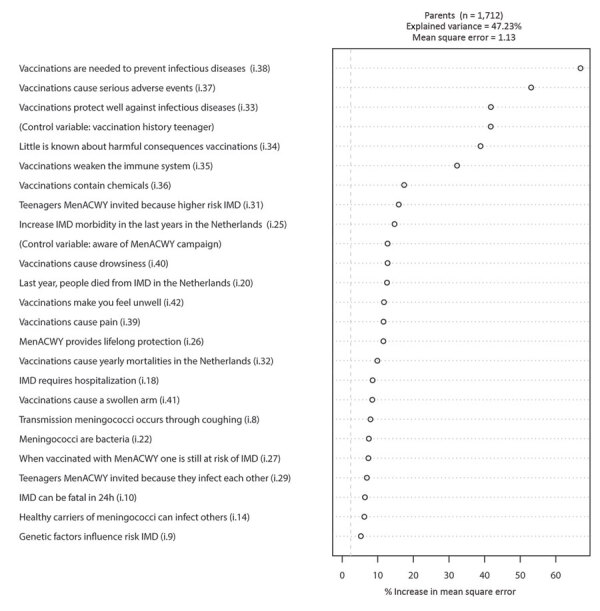
Variable importance ranking among parents in study of vaccination intent regarding IMD caused by *Neisseria meningitidis* strain W135, the Netherlands, 2018–2019. The 25 strongest predictors (i.e., knowledge and belief items [Table] and control variables), are ranked top to bottom, based on their ability to predict parental meningococcal conjugate [MenACWY] vaccination intention. Control variables are age, sex, education, income, region, social class, region of residence, vaccination record of the teenager, whether the respondent was aware of the MenACWY vaccination campaign, and whether teenagers and their parents were part of the first (cohorts 2004–2005) or the second MenACWY vaccination target group (cohorts 2001–2003). IMD, invasive meningococcal disease.

In the RF model for parents, 5 knowledge/belief items (plus the control variable “vaccination history teenager”) are considerably stronger predictors of MenACWY vaccination intention than the other items (Figure 1). Each of these items represents a belief regarding vaccines. The item “Vaccinations are needed to prevent infectious diseases” is the strongest predictor in this model (CVE 25.8%, MM 4.33–4.90). Note that when all other variables are kept constant, the lowest value for “Vaccinations are needed to prevent infectious diseases” (0) corresponds to a mean MenACWY vaccination intention of 4.33 and the highest value (4) corresponds to a mean MenACWY vaccination intention of 4.90.

The next items were “Vaccination can lead to various severe health conditions” (CVE 40.0%, MM 5.12–4.77), “Vaccinations protect well against infectious diseases” (CVE 42.2%, MM 4.54–5.08), “Little is known about the possible harmful consequences of vaccination” (CVE 46.4%, following the 45.25% CVE of “vaccination history teenager,” MM 5.00–4.57), and “Vaccinations weaken the immune system” (CVE 47.3%, MM 5.04–4.67). 

**Figure 2:**
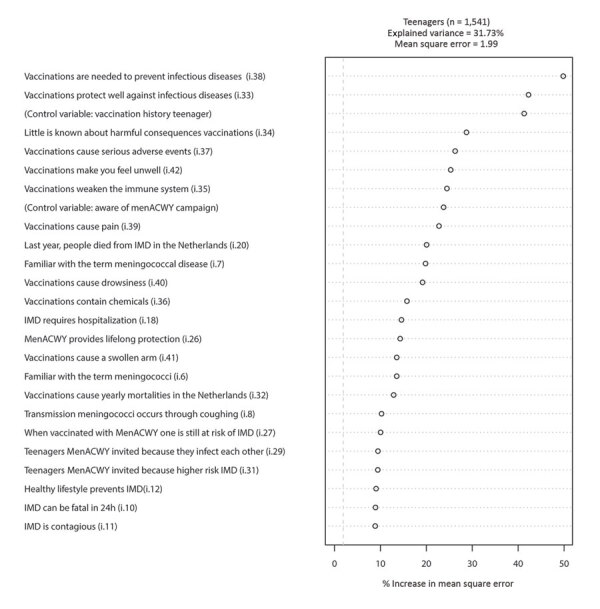
Variable importance ranking among teenagers in study of vaccination intent regarding IMD caused by *Neisseria meningitidis* strain W135, the Netherlands, 2018–2019. The 25 strongest predictors (i.e., knowledge and belief items [Table] and control variables) are ranked top to bottom, based on their ability to predict meningococcal conjugate (MenACWY) vaccination intention among teenagers. Control variables are age, sex, education, income, region, social class, region of residence, vaccination record of the teenager, whether the respondent was aware of the MenACWY vaccination campaign, and whether teenagers and their parents were part of the first (cohorts 2004–2005) or second MenACWY vaccination target group (cohorts 2001–2003). IMD, invasive meningococcal disease.

**Figure 3:**
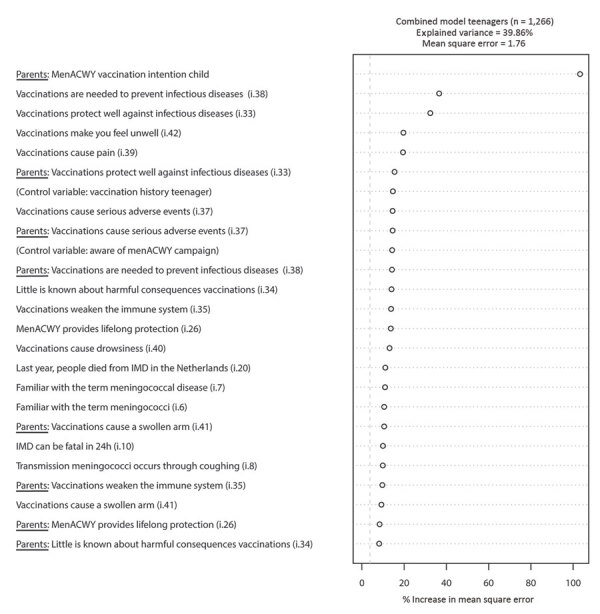
Variable importance ranking among teenagers (combined model) in study of vaccination intent regarding IMD caused by *Neisseria meningitidis* strain W135, the Netherlands, 2018–2019. The 25 strongest predictors (i.e., knowledge and belief items [Table] and control variables) are ranked top to bottom, based on their ability to predict meningococcal conjugate (MenACWY) vaccination intention among teenagers with a parent in the sample. This model includes both the knowledge and beliefs (Table) of teenagers, as well as the knowledge, beliefs, and MenACWY vaccination intention of their parents and the control variables from both groups as independent variables. Control variables are age, sex, education, income, region, social class, region of residence, vaccination record of the teenager, whether the respondent was aware of the MenACWY vaccination campaign, and whether teenagers and their parents were part of the first MenACWY vaccination target group (cohorts 2004–2005) or the second MenACWY vaccination target group (cohorts 2001–2003). IMD, invasive meningococcal disease.

In the model for teenagers (Figure 2), only 2 knowledge and beliefs items (plus the control variable “vaccination history teenager”) have a stronger ability to predict MenACWY vaccination intention than the other items, namely, “Vaccinations are needed to prevent infectious diseases” (CVE 16.7%, MM 3.66–4.61) and “Vaccinations protect well against infectious diseases” (CVE 18.7%, MM 4.00–4.62). In the combined model for teenagers (Figure 3), only 1 considerably strong predictor was observed, namely, the MenACWY vaccination intention of the parent (CVE 29.6%, MM 3.00–4.69).

## Discussion

Our study provides insights into MenACWY vaccination intentions and underlying knowledge and beliefs among teenagers and their parents at the start of the 2018 catch-up vaccination campaign in the Netherlands. Our study shows that teenagers were generally inclined to receive the MenACWY vaccination and parents were generally inclined to have their teenagers vaccinated. Both groups seemed aware of the severity and contagiousness of IMD, but we also identified knowledge gaps and misbeliefs. Knowledge and beliefs concerning the effectiveness of, need for, and safety of vaccines were the strongest predictors of MenACWY vaccination intentions. For teenagers, the strongest predictor of their own vaccination intention was whether their parent(s) wanted them to be vaccinated.

Although our study showed that MenACWY vaccination intentions among teenagers and their parents were relatively high, which is also reflected in the (preliminary) MenACWY vaccination uptake of 84% among teenagers (*35*), our study also revealed knowledge gaps and misconceptions concerning IMD, the MenACWY vaccination, and vaccinations in general. We observed differences in familiarity with various terms used to indicate IMD. Although the respondents were generally familiar with Dutch lay terms for the medical conditions caused by meningococci, few were familiar with the scientific terms for meningococci and meningococcal disease, despite the fact that these latter terms were mainly used in the communication materials. Furthermore, we found misbeliefs about the safety of vaccines. For example, we found a relatively strong agreement in our study population for the misbelief that vaccines annually cause the death of several children in the Netherlands. Nevertheless, we did find that teenagers and parents generally believed that IMD is a serious and contagious disease and that vaccinations are effective, safe, and needed.

Our results additionally show which specific misbeliefs and knowledge gaps might be prioritized to increase vaccination willingness among teenagers and parents. Teenagers and parents in our study who thought that vaccinations do not offer good protection against infectious diseases and that vaccinations are not necessary to prevent infectious diseases were less willing to accept MenACWY vaccination than were those who did not harbor these beliefs. In addition, vaccination intentions were lower among parents who believed that little is known about the possible harmful consequences of vaccination, that vaccinations weaken the immune system, and that vaccination can lead to serious adverse events. These beliefs about vaccinations in general surpassed all other knowledge and beliefs in their ability to predict MenACWY vaccination intentions. Previous studies have also found an influence of beliefs about the safety and effectiveness of vaccines on meningococcal vaccination decisions, both with regard to vaccines in general and with regard to the specific vaccine (*9*–*11*).

Of note, we did not find a major role for knowledge and beliefs associated with the severity of IMD in the variation of vaccination intentions, although previous research demonstrated that severity was an important factor in decisions for vaccination against IMD (*4*,*9*,*11*). One possible explanation for this finding is that the severity of IMD is a reason for persons to get vaccinated but does not explain why they do not intend to get vaccinated (*9*,*11*). Those persons, whose intention to get vaccinated is lower than that of most persons, provide variance in the dependent variable (MenACWY vaccination intention), and this variance is best explained by knowledge and beliefs about vaccinations in general.

Our results further show that parents were more willing to have their teenagers vaccinated than were the teenagers themselves and that parents were somewhat more knowledgeable about IMD and the MenACWY vaccination. It has been argued that teenagers are less knowledgeable about health issues than adults because, among other things, teenagers have had less contact with health issues and the healthcare system (*36**,**37*). Similarly, teenagers are likely to have limited (direct or indirect) experience with IMD, whereas their parents are more likely to recall the 1999–2002 outbreak of IMD in the Netherlands, caused by group C meningococci (*38*).

The lower vaccination intentions, knowledge gaps, and misbeliefs among teenagers might suggest that, to achieve high vaccine uptake during emerging outbreaks, public health authorities should focus on risk and benefits communication about vaccine-preventable diseases and vaccines for teenagers. However, our results also indicate that teenagers’ willingness to adopt vaccination is most strongly predicted by their parents’ willingness to have their child vaccinated. In this light, we need to consider whether intensifying the communication for teenagers would indeed be of much help for increasing their vaccine uptake. More effective might be filling the knowledge gaps and debunking misbeliefs that underlie parental vaccination intentions. Nevertheless, the observed predictive ability of parental vaccination intention does not necessarily imply that parents decide whether their teenager should be vaccinated. It probably also reflects the commonalities in knowledge, beliefs, and norms in social groups or networks (*39*).

Our study has some limitations. First, questions from our survey were developed specifically for this population and disease and are therefore not directly applicable to study knowledge and beliefs in different population or disease contexts. Nevertheless, we believe that gaining these insights about specific knowledge and beliefs that influence vaccination decisions can provide more valuable input for communication strategies than can survey studies that assess perceptions of risk with more general constructs. Second, our study focused solely on knowledge and beliefs and their role in vaccination intentions. We did not include in our research other predictors of health behavior (e.g., the influence of subjective norm perceptions and self-efficacy perceptions [*20*]). Last, although our study population was sampled to be representative of the larger population and the response rate was relatively high, because participation was voluntary, the final selection of participants might include more persons with a specific interest in the topic.

For future communications accompanying vaccination campaigns combating outbreaks, we recommend concentrating on filling knowledge gaps and addressing specific misbeliefs about the effectiveness and safety of vaccines. In addition, communicators should pay attention to the wording of the messages, which should ideally correspond to the lay vocabulary. As for teenagers, the strongest predictor of their own willingness to be vaccinated was their parents’ vaccination intention. We therefore suggest that parents remain a target group in communications about vaccination of teenagers.

Appendix Survey used in study of meningococcal W135 disease vaccination intent, the Netherlands, 2018–2019.
