# Supplementary material for: Meningococcal W135 Disease Vaccination Intent, the Netherlands, 2018–2019
Source: Emerg Infect Dis. 2020 Jul;26(7):1420–9. doi: 10.3201/eid2607.191812 (PMC7323551; doi:10.3201/eid2607.191812)
Supplement: Appendix — Survey used in study of meningococcal W135 disease vaccination intent, the Netherlands, 2018–2019. [file 19-1812-Techapp-s1.pdf]

Article DOI: https://doi.org/10.3201/eid2607.191812

Meningococcal W135 Disease Vaccination Intent, the Netherlands, 2018–2019

Appendix

Appendix Table. Survey questions and items addressing knowledge and beliefs about meningococcal disease (questions A-B, items 1-25), the menACWY vaccination (question C, items 26-31), and vaccinations in general (questions D-E, items 32-42), descriptive statistics (frequencies (no.) and percentages (%) / means (M) and standard deviations (SD)) in parents and teenagers, and P-values from chi2 tests and independent t-tests* indicating differences between parents and teenagers

Survey question (A-E) and survey item (1-42)	Summarized item (corresponding to the Figures)	Truthfulness of item (Item source)†	No. (%) Parents	No. (%) Teenagers	P-value chi^2 test
A. Have you ever heard about… (scale:1-3) §		-	-	-	-
1. “nekkramp” (= Dutch lay term for meningitis)	Familiar with the Dutch lay term for meningitis (*nekkramp)*	-	1. 1184 (66.4)<br>2. 542 (30.4)<br>3. 58 (3.3)	293 (18.3)<br>802 (50.0)<br>508 (31.7)	<0.001
2. “hersenvliesontsteking” (= Dutch lay term for meningitis)	Familiar with the Dutch lay term for meningitis *(hersenvliesontsteking)*	-	1. 1371 (76.8)<br>2. 407 (22.8)<br>3. 6 (0.3)	414 (25.8)<br>911 (56.8)<br>278 (17.3)	<0.001
3. “bloedvergiftiging”(=Dutch lay term for septicemia)	Familiar with the Dutch lay term for septicemia *(bloedvergiftiging)*	-	1. 1389 (77.9)<br>2. 390 (21.9)<br>3. 5 (0.3)	604 (37.7)<br>804 (50.2)<br>195 (12.2)	<0.001
4. Meningitis	Familiar with the term meningitis	-	1. 883 (49.5)<br>2. 756 (42.4)<br>3. 145 (8.1)	103 (6.4)<br>358 (22.3)<br>1142 (71.2)	<0.001
5. Septicemia	Familiar with the term septicemia	-	1. 548 (30.7)<br>2. 521 (29.2)<br>3. 715 (40.1)	57 (3.6)<br>150 (9.4)<br>1396 (87.1)	<0.001
6. Meningococci	Familiar with the term meningococci	-	1. 1044 (58.5)<br>2. 718 (40.2)<br>3. 22 (1.2)	243 (15.2)<br>771 (48.1)<br>589 (36.7)	<0.001
7. Meningococcal disease	Familiar with the term meningococcal disease	-	1. 819 (45.9)<br>2. 827 (46.4)<br>3. 138 (7.7)	194 (12.1)<br>689 (43.0)<br>720 (44.9)	<0.001
B. You will be shown a number of statements about meningococcal disease. Please indicate to what extent you think the statements are true or false. (scale 0-4) ‡		Truthfulness of item (Item source)†	M (SD) Parents	M (SD) Teenagers	P-value t-test
8. One can get infected with meningococci if someone nearby coughs or sneezes	Transmission meningococci occurs through coughing	T (si)	2.4 (1.1)	2.2 (1.0)	<0.001
9. Genetic factors influence the risk of meningococcal disease	Genetic factors influence risk IMD	F (ei)	1.6 (0.9)	1.9 (0.9)	<0.001
10. Meningococcal disease can be fatal within 24 hours from onset	IMD can be fatal in 24h	T (si)	2.8 (1.0)	2.3 (1.0)	<0.001
11. Meningococcal disease is contagious	IMD is contagious	T (si)	2.9 (1.0)	2.7 (0.9)	<0.001
12. A healthy lifestyle prevents meningococcal disease	Healthy lifestyle prevents IMD	F (ei)	1.5 (1.0)	1.9 (1.0)	<0.001
13. One can carry meningococci in the nose and throat without becoming sick	Carriage without illness is possible	T (si)	2.8 (0.9)	2.3 (0.8)	<0.001
14. Healthy carriers of meningococci can infect others	Healthy carriers can infect others	T (si)	2.7 (0.9)	2.2 (0.9)	<0.001
15. Meningococcal disease can lead to mental or physical impairments	IMD causes impairments	T (si)	3.0 (0.9)	2.5 (0.9)	<0.001

Survey question (A-E) and survey item (1-42)	Summarized item (corresponding to the Figures)	Truthfulness of item (Item source)†	No. (%) Parents	No. (%) Teenagers	P-value chi^2 test
16. Certain hormones increase the risk at meningococcal disease	Certain hormones increase risk IMD	F (ei)	2.1 (0.7)	2.1 (0.6)	0.614
17. If you take medicines against meningococcal disease, you will become healthy again	Medicines cure IMD	N (si)	2.4 (0.9)	2.4 (0.8)	0.037
18. Meningococcal disease requires hospitalization for treatment	IMD requires hospitalization	T (si)	3.4 (0.7)	3.1 (0.8)	<0.001
19. A weak immune system increases the risk of meningococcal disease	Weak immune system increases risk IMD	F (ei)	3.1 (0.7)	2.9 (0.8)	<0.001
20. In the past year, people died from meningococcal disease in the Netherlands	Last year, people died from IMD in the Netherlands	T (si)	3.6 (0.7)	3.1 (0.9)	<0.001
21. One can get infected with meningococci by kissing someone	Transmission meningococci occurs through kissing	T (si)	2.5 (1.0)	2.4 (0.9)	0.019
22. Meningococci are bacteria	Meningococci are bacteria	T (si)	3.0 (1.0)	2.8 (0.8)	<0.001
23. Adults can contract meningococcal disease	Adults are at risk of IMD	T (si)	3.3 (0.8)	3.0 (0.8)	<0.001
24. One can prevent meningococcal disease with thorough handwashing	Washing hands prevents IMD	F (ei)	1.6 (1.0)	1.9 (0.9)	<0.001
25. In the past couple of years, more people in the Netherlands fell ill due to one of the meningococcus types	Increase IMD morbidity in the last years in the Netherlands	T (si)	3.2 (0.8)	2.7 (0.8)	<0.001
C. You will be shown a number of statements about the vaccination against meningococcal disease type A, C, W and Y. Please indicate to what extent you think the statements are true or false. (scale 0-4) ‡		-	-	-	-
26. The meningococcal vaccination provides lifelong protection against meningococcal disease	MenACWY provides lifelong protection	F (ei)	2.0 (0.9)	2.1 (0.9)	0.079
27. Even while one is vaccinated against meningococci, one can still contract meningococcal disease	When vaccinated with menACWY one is still at risk of IMD	T (si)	2.3 (0.9)	2.1 (1.0)	<0.001
28. Teenagers are invited for the meningococcal vaccination because they are still young	Teenagers menACWY invited because they are young	N (ei)	2.3 (1.1)	2.5 (0.9)	<0.001
29. Teenagers are invited for the meningococcal vaccination because they can easily infect each other with meningococcus	Teenagers menACWY invited because they infect each other	T (si)	2.7 (1.0)	2.6 (0.8)	<0.001
30. Teenagers are invited for the meningococcal vaccination because they are still growing	Teenagers menACWY invited because they are still growing	F (ei)	2.0 (1.0)	2.2 (0.9)	<0.001
31. Teenagers are invited for the meningococcal vaccination because they are more at risk of meningococcal disease	Teenagers menACWY invited because higher risk IMD	T (ei/si)	3.0 (0.8)	2.7 (0.8)	<0.001
D. You will be shown a number of statements about vaccinations in general. Please indicate to what extent you think the statements are true or false. (scale 0-4)‡		-	-	-	-
32. Every year, a number of children in the Netherlands die from the harmful consequences of vaccinations	Vaccinations cause yearly mortalities in the Netherlands	F (ei/vsw)	2.3 (1.2)	2.1 (1.0)	<0.001
33. Vaccinations protect well against infectious diseases	Vaccinations protect well against infectious diseases	T (ei/si)	3.1 (0.8)	2.9 (0.8)	<0.001
34. Little is known about the possible harmful consequences of vaccination	Little known about harmful consequences vaccinations	F (vsw)	1.9 (1.2)	2.0 (0.9)	<0.001
35. Vaccinations weaken the immune system	Vaccinations weaken the immune system	F (vsw)	1.5 (1.1)	1.7 (1.0)	<0.001

Survey question (A-E) and survey item (1-42)	Summarized item (corresponding to the Figures)	Truthfulness of item (Item source)†	No. (%) Parents	No. (%) Teenagers	P-value chi^2 test
36. Vaccinations contain chemicals	Vaccinations contain chemicals	T (vsw)	2.6 (1.0)	2.5 (0.9)	<0.001
37. Vaccination can lead to various severe health conditions	Vaccinations cause serious adverse events	N (vsw)	1.9 (1.1)	1.8 (0.9)	0.020
38. Vaccinations are needed to prevent infectious diseases	Vaccinations are needed to prevent infectious diseases	T (vsw)	3.3 (0.9)	3.0 (0.8)	<0.001
E. Please read the following statements and indicate what you think of these statements. (scale 0-6) ‡		-	-	-	-
39. A vaccination…	Vaccinations cause pain	N (ei)	2.3 (1.4)	2.8 (1.7)	<0.001
40. After a vaccination you feel tired or drowsy…	Vaccinations cause drowsiness	N (ei)	2.5 (1.3)	2.5 (1.6)	0.410
41. After a vaccination in your arm, your arm swells…	Vaccinations cause a swollen arm	N (ei)	2.3 (1.3)	2.1 (1.7)	<0.001
42. After a vaccination, you become unwell…	Vaccinations make you feel unwell	N (ei)	2.5 (1.2)	2.5 (1.7)	0.462

*Additional paired *t*-tests were performed for the 1318 pairs of teenagers and parents from the same household. The results from these tests yielded, to a large extent, similar results as those from the independent t-tests.
† Truthfulness of item: T=true, F=false, N=neither true or false (depends on interpretation);
Item source: si = scientific information, ei = explorative interviews, vsw = vaccine-skeptical websites
‡ Answer categories items 1-7:
1 (Yes, I have heard about it and know what it is),
2 (Yes, I have heard about it, but do not know exactly what it is),
3 (No, I have never heard about it);
Answer categories items 8-38: 0 (certainly not true), 1 (probably not true), 2 (don't know), 3 (probably true), 4 (certainly true);
Answer categories item 39: 0 (does not hurt at all) – 6 (hurts a lot);
Answer categories items 40-42**:** 0 (never) – 6 (always)
